# Paradigm Shift in Rhinoplasty with Virtual 3D Surgery Software and 3D Printing Technology

**DOI:** 10.1055/a-2272-5273

**Published:** 2024-04-10

**Authors:** Man Koon Suh, Joo-Yun Won, Jung-Hwan Baek

**Affiliations:** 1 JW Plastic Surgery Center, Gangnam-gu, Seoul, Republic of Korea; 2 Clinical and Translational Research Institute, Anymedi Inc., Seoul, South Korea; 3 H Plastic Surgery Clinic 5F, Seocho-gu, Seoul, Republic of Korea

**Keywords:** rhinoplasty, virtual 3D plastic surgery software, customized nasal implant, 3D printing, artificial intelligence

## Abstract

Most Asians have a nose with a short columella and a low dorsum; augmentation rhinoplasty using implants is commonly performed in Asian countries to achieve a taller and more well-defined nasal dorsum. However, the current knowledge is insufficient to fully understand the various subjective desires of patients, reflect on them during surgery, or to objectively analyze the results after surgery. Advances in digital imaging technologies, such as 3D printing and 3D scanning, have transformed the medical system from hospital-centric to patient-centric throughout the medical field. In this study, we applied these techniques to rhinoplasty. First, we used virtual 3D plastic surgery software to enable surgical planning through objectified numerical calculations based on the visualized data of the patient's medical images rather than simple virtual plastic surgery. Second, the customized nasal implant was manufactured by reflecting the patient's anatomical shape and virtual 3D plastic surgery data. Taken together, we describe the surgical results of applying these rhinoplasty solutions in four patients. Our experience indicates that high fidelity and patient satisfaction can be achieved by applying these techniques.

## Introduction

Rhinoplasty is generally regarded as a plastic surgery technique with a high rate of revision surgery. 1 Because the nose is located at the center of the face, it influences the overall visual impression of the individual. If the surgery does not consider the overall balance of the face, the surgical result does not come out as desired, and reoperation should be considered. To solve this problem, various methods to show the postoperative appearance have been proposed; however, the accuracy of predicting the surgical result through unobjective pictures or drawings is low.

Silicone implants, which are most commonly used for rhinoplasty, have been developed in the form of semifinished precarved implants using the initial block carving method. 2 3 However, reflecting the various anatomical differences and needs of each patient, silicone implants are being manufactured in various types and thicknesses. Compared with the previous silicon block engraving method, currently available silicone implants have many advantages, including shortening the operation time and overcoming the proficiency of prosthetic sculpting; however, this technology is insufficient to produce a perfectly fitting implant.

The development of medical imaging technology and 3D printing technology has brought about a change in the medical field toward patient-centered, patient-customized implants. 4 5 6 To apply this technology to rhinoplasty, virtual 3D plastic surgery software can be used to extract data regarding the patient's skin, bone, and cartilage shapes from 3D computed tomography (CT) images. This software can then be used to create a customized nasal implant using 3D printing technology. First, the patient creates the desired nose shape from their current face based on the patient's surface 3D CT image implemented through virtual 3D plastic surgery software. Based on this, the surgeon confirms the patient's requirements and establishes a surgical plan by discussing with the patient, considering their conditions, such as skin thickness and capsules, in case of reoperation. According to the established surgical plan, the developer designs a patient-specific implant using artificial intelligence-powered specialized software and manufactures it using 3D modeling and 3D printing technology. Finally, the surgeon performs the operation according to a virtual plastic-based surgical plan. In this study, we describe the results of several cases in which virtual plastic surgery, objective 3D modeling design, and 3D printing-based customized implants were applied to achieve each patient's individual desire.

## Idea

**Fig. 1:**
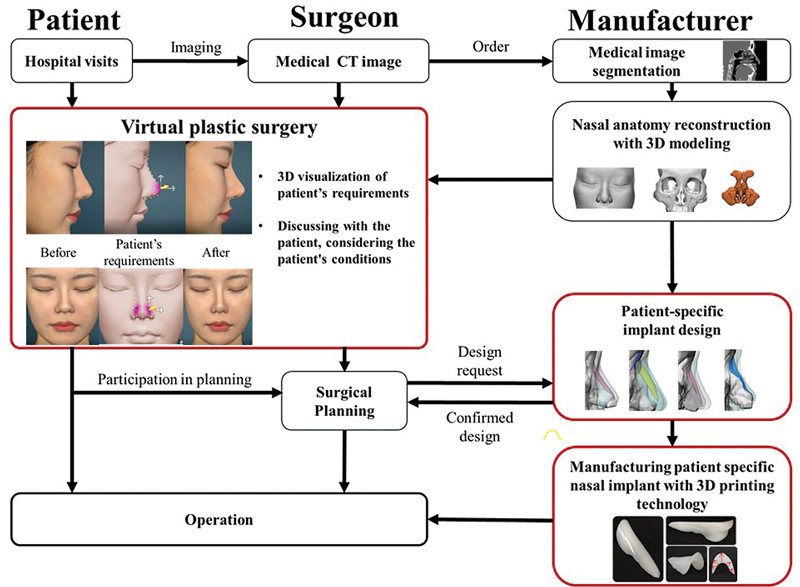
Flowchart showing the process of customized 3D printing implant manufacturing based on virtual plastic surgery. CT, computed tomography.

The production of customized implants based on virtual plastic surgery reflecting the needs of the patient involves the harmonization of the patient's requirements with the surgical plan of the surgeon. This requires three-way communication between the patient, surgeon, and implant manufacturer ( Fig. 1 ). This protocol has been extensively explained in previous studies. 7 8 In brief, a medical image is obtained through a CT scan from a patient who visits the hospital for rhinoplasty. The manufacturer uploads the patient medical image to the Materialize Mimics software (Materialize, Leuven, Belgium) and adjusts the Hounsfield Units value to divide the data into the specific part of the face, including the skin, bones of the region including the nasal bone and the orbital bone, and the nasal cavity; Snake and U-net deep learning algorithms can be used for this division. Most medical images do not have a quality sufficient for segmenting nasal cartilages. Manufacturers predict nasal cartilage by applying deep learning technology and commonly known anatomical information such as airway and thickness of nasal septum cartilage. Anatomically, the nasal bone and nasal cartilage are connected at the same height, and the shape of the nasal cartilage is a thin membrane of approximately 0.5 mm, similar to the shape of the edge of the nasal cavity. Therefore, nasal cartilage can be predicted by applying an offset to the segmented image so that the nasal cavity image matches the height of the nasal bone. The part to be attached to the implant is modeled according to the 3D-modeled nose shape, and a surgical plan such as length extension is applied. Based on the virtual plastic surgery that reflects the patient's request, the final surgical plan between the patient and the surgeon is completed to model the appearance (volume and 3D shape) of the nose implant. A 3D printer makes a mold to produce a nasal implant that has been confirmed by the surgeon. By injecting medical-grade silicone (Nusil Technology LLC, Carpinteria, CA), which has been approved as safe by the Food and Drug Administration, into the mold, a customized nasal implant that can be implanted in a patient is manufactured.

### Cases

This study shows representative cases among patients who applied virtual plastic surgery and patient-specific implants from April 2020 to December 2022. Table 1 describes the cases.

**Table 1:** Case description

Case	Gender	Operation	Patient requirements
**1**	Female	Primary	Correction of low dorsum and upturned tip
**2**	Female	Reoperation	Correction of implant deviation due to asymmetry of nasal bone and upper lateral cartilage
**3**	Male	Primary	Radix augmentation
**4**	Male	Primary	Narrowing of the broad bony vault and augmentation of the nasofrontal angle Nasal aesthetic line and natural nasal hump Nasal projection and minimal nasal tip lengthening

### Case 1

**Fig. 2:**
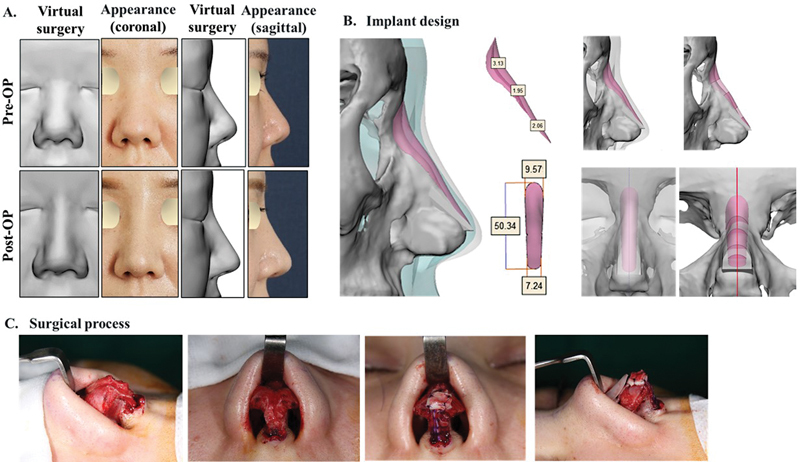
Case 1: A patient with a low dorsum and upturned tip who underwent dorsal augmentation tip plasty. The patient's request was reflected in virtual plastic surgery, and the postoperative surgical result has the exact same dorsal height and curvature from the side view as shown in simulation surgery. ( **A** ) Virtual plastic surgery and actual appearance before and after surgery, ( **B** ) customized implant design, and ( **C** ) surgical process.

This patient was a primary augmentation rhinoplasty case who required correction of the low dorsum and upturned tip. The patient expressed the desired dorsal height and line curvature from the side view before surgery through virtual plastic surgery software ( Fig. 2A ). After discussing the results of the virtual plastic surgery with the surgeon and establishing a final surgical plan, a patient-specific nasal implant was manufactured ( Fig. 2B ). Nasal dorsal augmentation was performed with a customized nasal implant according to the surgical plan. For tip plasty, a columellar strut with conchal cartilage, spanning suture of the lateral crura, derotation graft with conchal cartilage, and conchal cartilage onlay graft on the tip were applied. Comparison of the preoperative and postoperative results of the virtual plastic surgery and patient appearance in the sagittal and coronal directions ( Fig. 2 ) confirmed that the postoperative result was the same as that of the virtual plastic surgery.

One of the biggest advantages of customized nasal implants is the ability to confirm the patient's wishes through virtual plastic surgery software and to implement them through surgery, which can minimize the patient's subjective dissatisfaction.

### Case 2

**Fig. 3:**
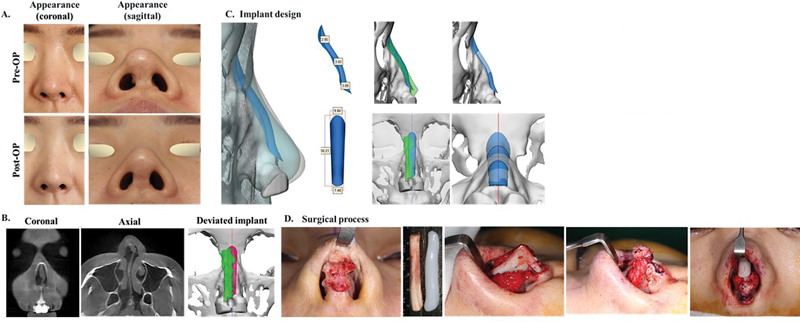
Revisional Case 2: The patient showed a good result in correcting implant deviation with a customized implant ( **A** ) actual appearance before and after surgery, ( **B** ) medical CT image, ( **C** ) customized implant design, and ( **D** ) surgical process.

Patient had previously undergone dorsal augmentation with a GoreTex implant and tip plasty using septal cartilage. The patient visited the clinic for correction of implant deviation due to asymmetry of the nasal bone and upper lateral cartilage and correction of an unfavorable tip shape and septal collapse ( Fig. 3A, B ). A patient-specific nasal implant was fabricated according to the anatomical shape of the asymmetric nasal bone ( Fig. 3C ). For tip revision and mid-vault reconstruction, bilateral septal extension grafts (tongue-and-groove type) with costal cartilage and bilateral alar rim grafts with costal cartilage were performed ( Fig. 3D ). The deviated implant was well-corrected with a 3D printed patient-specific implant technique. Implant deviation is a common complication of nasal dorsal augmentation and is generally caused by asymmetry of the nasal bone and upper lateral cartilage. Since the customized nasal implant is manufactured by reflecting the asymmetry of the nasal bone, the possibility of implant deviation is estimated to be minimized; therefore, the correction of deviated implants is believed to be effective in secondary rhinoplasty.

### Case 3

**Fig. 4:**
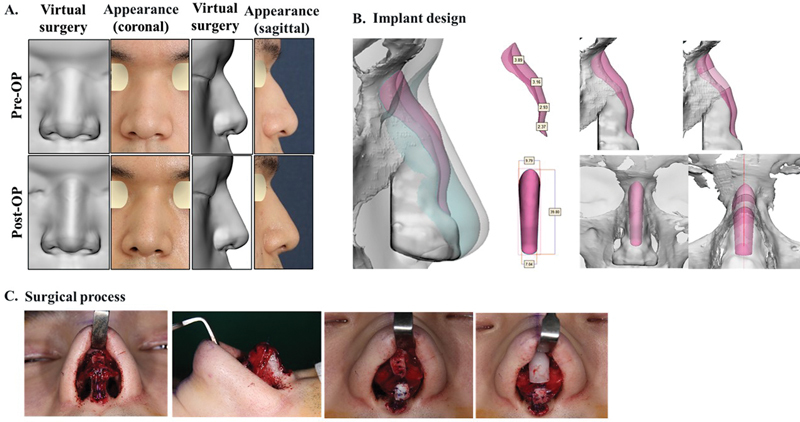
Case 3: A male patient with an acute-angled radix who wished to undergo dorsal augmentation and tip plasty to achieve a straight line from the radix to the tip, as preferred by male patients. ( **A** ) Virtual plastic surgery and actual appearance before and after surgery, ( **B** ) customized implant design, and ( **C** ) surgical process.

Case 3 was a male patient with acute and deep radix; it is difficult to fabricate an implant for dorsal augmentation in such patients as most readymade implants do not exactly fit this shape of the radix, and a dead space tends to occur between the implant and the nasal bone, leading to thick capsule formation. In addition, the radix tends to have a rounded shape rather than a straight line from the side view preferred by men. To overcome this difficulty, dorsal augmentation with a customized nasal implant and tip plasty containing a columella strut with conchal cartilage and conchal cartilage onlay graft on the tip was performed on this patient. After surgery with radix augmentation, a straight line from the side view was formed ( Fig. 4 ).

### Case 4

**Fig. 5:**
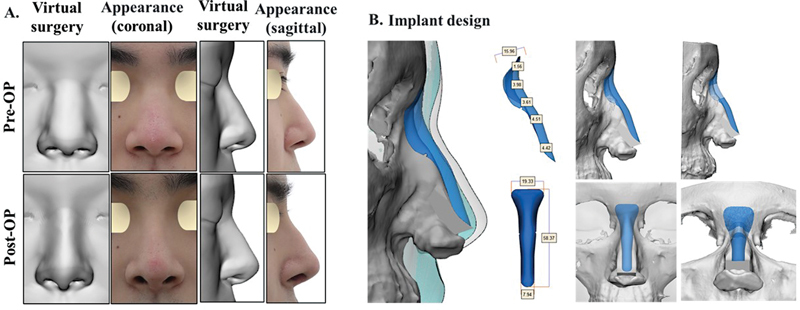
Case 4: A male patient who requested to maintain the aesthetic line and nasal hump preferred by male patients. ( **A** ) Virtual plastic surgery and actual appearance before and after surgery and ( **B** ) customized implant design.

Case 4 was a male patient with primary rhinoplasty who desired narrowing of the broad bony vault and augmentation of the nasofrontal angle while simultaneously seeking a nasal aesthetic line and a natural nasal hump. Additionally, nasal projection and minimal lengthening of the nasal tip were requested. These requirements were reflected in the virtual plastic surgery, emphasizing the narrowing of the nasal aesthetic line and mid-vault ( Fig. 5A ). To reflect the needs of the patient, we designed a patient-specific nasal implant considering the following six points. First, the implant was designed up to the top of the nasion for the nasofrontal line, and for the nasal aesthetic line, the seagull pattern was applied to the cephalic area of the implant. Second, the hump design of the dorsal side of the implant was completed for the natural hump line. Third, the position of the alar cartilage was modified with consideration of the septal extension graft, and the basal side of the implant was designed to match the 3D shape of the nasal bone and the upper lateral cartilage. Fourth, the caudal part of the implant was modified to be thin on the basal side, in consideration of the septal extension graft ( Fig. 5B ). Surgery was performed with wide dissection to include the nasofrontal suture line, and intranasal lateral and medial osteotomy was performed to create a narrowing of the bony vault. A septal extension graft and columella strut graft were performed using the rib cartilage. After inserting the customized nasal implant and confirming the correct position, the tip onlay graft was completed, and skin and mucosal sutures were performed.

## Discussion

Rhinoplasty has a higher rate of revision when performed in Asian patients. The main causes are anatomically thick skin, the small and weak structure of the bone and cartilage framework compared with Caucasian noses, and more importantly, improper use of dorsal implants. In clinical practice, if the patient's request is ambiguous or unrealistic, patients often experience dissatisfaction with the surgical results. 9

To overcome these anatomical limitations, many surgeons have grafted various materials and applied various surgical techniques. Silicone implants are inexpensive and safe materials when used properly and are widely used to augment the low nasal dorsum in Asian countries. The readymade silicone implant is easy to carve, but the undersurface that contacts the nasal bone and upper lateral cartilages does not accurately implement the surface contour of the patient's nasal bone and upper lateral cartilages; therefore, it does not fit into the irregular and asymmetric contour of the dorsum properly, creating a gap between the implant undersurface and nasal framework. This can lead to patient dissatisfaction due to various complications such as implant deviation or caudal slipping, thick capsule formation, seroma, and hematoma formation. For this reason, various studies were conducted to manufacture and apply a customized silicone nasal implant based on 3D printing technology. 10 11 12 It was reported that the anatomical shape of the patient's nose could be reproduced with 3D printing so that the curvature of the nasal bone and cartilage matched, thereby reducing the possibility of side effects such as deviation, dripping, or the development of thick gaps. However, these studies did not suggest a method to reflect the patient's exact needs, as they produced a patient CT-based customized nose implant.

**Fig. 6:**
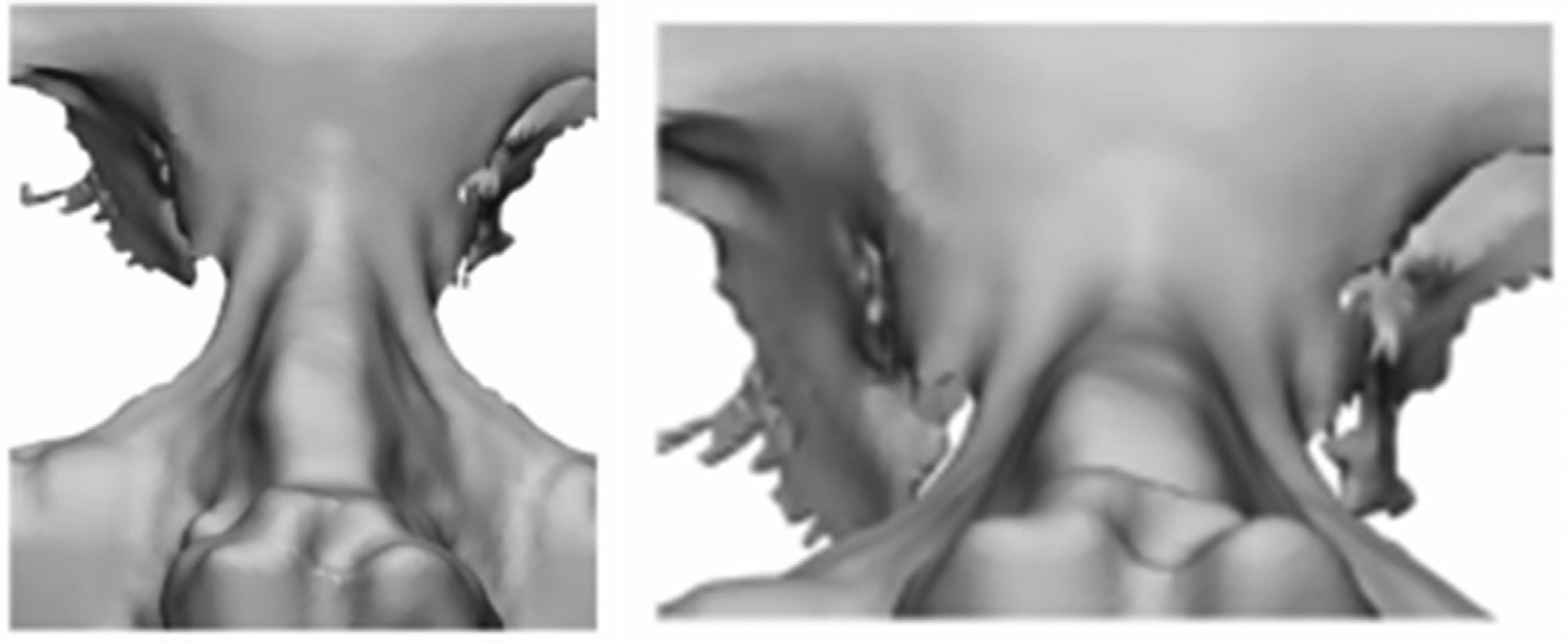
**T** he asymmetry of the nasal bone and upper lateral cartilage.

If the patient's wishes are accurately communicated to the surgeon and reflected in the operation, the results can be realized, the patient's postoperative satisfaction will increase, and the frequency of reoperations will consequently decrease. In the past, surgeons have suggested various methods (photos, drawings, 2D virtual plastic surgery programs, celebrity photos) when planning the surgery, but these methods are insufficient to accurately convey the intention between the patient and the operator. 13 14 To solve this problem, we applied data science that was objective and digitized from patient image scanning, virtual plastic surgery, implant production, and postoperative result analysis, as well as ideas and artificial intelligence software, to correct insufficient data in all processes. Using the same process as in Fig. 1 , the results of the virtual plastic surgery and the actual surgery were confirmed by applying a customized 3D nose implant made according to the surgical plan reflecting the patient's opinion. Through the patient cases, it was shown that the simulation in which the patient participated is practically feasible. There are many cases where the implants deviated due to the asymmetry of the nasal bone and upper lateral cartilage. However, the patient-customized implants were made to fit the asymmetry, so it was effective in correcting the deviated nose ( Figs. 3B and 6 ). In addition, in the recent increase in male plastic surgery, there was a great advantage in male-specific nose shaping such as radix, hump, and aesthetic lines ( Figs. 4 and 5 ). Certainly, for reasons such as CT precision, cartilage estimation, and soft tissue thickness following implant insertion, the surgical result could not be predicted perfectly, but an error of less than 1 mm was obtained through numerous data and artificial intelligence learning. If more data are accumulated in the future, the error is expected to be further reduced, and we are proceeding with a further study for long-term follow-up analysis.

In conclusion, this study is meaningful as an example of the actual application of data science and engineering technology in rhinoplasty in the medical field. The accumulation of such objective data can be used for postoperative evaluation and as a tool to verify new surgical methods or to analyze the results more objectively. Overall, shortening the operation time, potentially reducing the chance of complications, and increasing patient satisfaction can be expected to reduce the frequency of reoperation.
